# JAK inhibition and axial spondyloarthritis: new steps on the path to understanding pathophysiology

**DOI:** 10.3389/fimmu.2025.1488357

**Published:** 2025-03-04

**Authors:** Francesco Ciccia, Dennis McGonagle, Ranjeny Thomas, Helena Marzo-Ortega, David A. Martin, Arne Yndestad, Mikhail Volkov

**Affiliations:** ^1^ Dipartimento di Medicina di Precisione, Università della Campania L. Vanvitelli, Naples, Italy; ^2^ Leeds Institute of Rheumatic and Musculoskeletal Medicine, University of Leeds and NIHR Leeds Biomedical Research Centre, The Leeds Teaching Hospitals NHS Trust, Leeds, United Kingdom; ^3^ Frazer Institute, The University of Queensland, Woolloongabba, QLD, Australia; ^4^ Pfizer Inc, Cambridge, MA, United States; ^5^ Pfizer Inc, Oslo, Norway; ^6^ Pfizer BV, Capelle aan den IJssel, Netherlands

**Keywords:** Axial spondyloarthritis, gut-joint axis, JAK-STAT pathway, Janus kinase inhibitor, pathophysiology

## Abstract

Axial spondyloarthritis (axSpA) is a chronic inflammatory disease that predominantly affects the sacroiliac joints and spine. Tumor necrosis factor (TNF) and interleukin (IL)-17A are key cytokines in disease pathogenesis and are established axSpA treatment targets. Recently, axSpA treatment options have been complemented by Janus kinase inhibitors (JAKi), which inhibit various cytokines without directly impacting TNF or IL-17 signaling. The effect of JAKi on axSpA remains under investigation: besides a JAK2-mediated (and potentially tyrosine kinase 2 [TYK2]-mediated) effect on the IL-23/IL-17 axis, emerging evidence suggests γδ T cells, type 3 innate lymphoid cells, and mucosa-associated invariant T cells, which are dependent on IL-7 and/or IL-15 and thus on JAK1, are strongly inhibited by JAKi used to treat axSpA. This review summarizes potential effects of JAKi on axSpA and shows evidence from pre-clinical/clinical studies. Greater understanding of the mechanisms of action of available treatments may improve knowledge of axSpA and pave the road for future therapies.

## 1 Introduction

Axial spondyloarthritis (axSpA) is a form of spondyloarthritis with predominant spinal involvement (1). Other differentiated or overlapping forms include psoriatic arthritis (PsA), reactive arthritis and enteropathic arthritis, associated with inflammatory bowel disease (IBD) (2). The main clinical characteristic of axSpA is chronic axial inflammation, affecting the spine and sacroiliac joints (1). Classification into non-radiographic axSpA and radiographic axSpA (r-axSpA), previously known as ankylosing spondylitis (AS), is based on the presence or absence of radiographically detectable sacroiliitis, with radiographic disease being, to a large degree, a reflection of chronicity (1).

Recent translational research has deepened our understanding of axSpA, focusing on immune cells and pathways. Treatment options have expanded with the introduction of biological and targeted synthetic biological (b) and targeted synthetic disease-modifying antirheumatic drugs (DMARDs), with prominent additions including tumor necrosis factor (TNF) and interleukin (IL)-17 inhibitors (3). Despite this, many patients with axSpA have ongoing disease activity and poor quality of life (4); some studies show that almost 40% of patients need to change treatment due to suboptimal disease control (5). Therefore, an unmet treatment need remains and the recent addition of Janus kinase inhibitors (JAKi) to the axSpA therapeutic armamentarium is welcome.

The efficacy of JAKi in tackling axSpA and PsA clinical manifestations has been shown in experimental models (6) and clinical studies (7–13). This is intriguing, as the JAK/signal transducer and activator of transcription (STAT) signaling pathway is not directly involved in either TNF or IL-17A, the two cardinal cytokines that have been successfully blocked in axSpA (14). A precise understanding of the mechanisms by which JAKi suppress axSpA disease activity is important, as this could pave the road for personalized medicine and tailored treatments for specific patient groups. This is pertinent as the efficacy of other treatments in spondyloarthritis varies depending on the organs affected. For example, IL-17 inhibitors have shown no efficacy in IBD (15), a common extra-musculoskeletal manifestation of axSpA, and IL-23 inhibitors do not work in the spine in r-axSpA (16). We present a state-of-the-art review of axSpA pathophysiology in the context of the mechanisms by which JAKi potentially exert their therapeutic effect.

## 2 An updated model of axSpA pathogenesis

The precise pathophysiology of axSpA is not fully understood, but likely encompasses a complex interaction between genetic risk factors, including human leukocyte antigen (HLA)-B27 and innate immune genes, biomechanical stress, and microbiome alterations (17). Disease onset occurs at sites associated with mechanical stress, including entheses, sacroiliac joints, and extra-musculoskeletal sites including the anterior uveal tract (2). The resulting microdamage is hypothesized to trigger ineffective repair in susceptible individuals, leading to chronic inflammation (2). AxSpA chronic inflammation leads to post-inflammatory remodeling and irreversible changes in affected tissues, resulting in new bone formation in the spine (17). The pathological processes leading to structural damage in axSpA can essentially be summarized in three phases: (1) inflammation, (2) variable bone erosion, and (3) post-inflammatory new bone formation (18).

**Figure 1:**
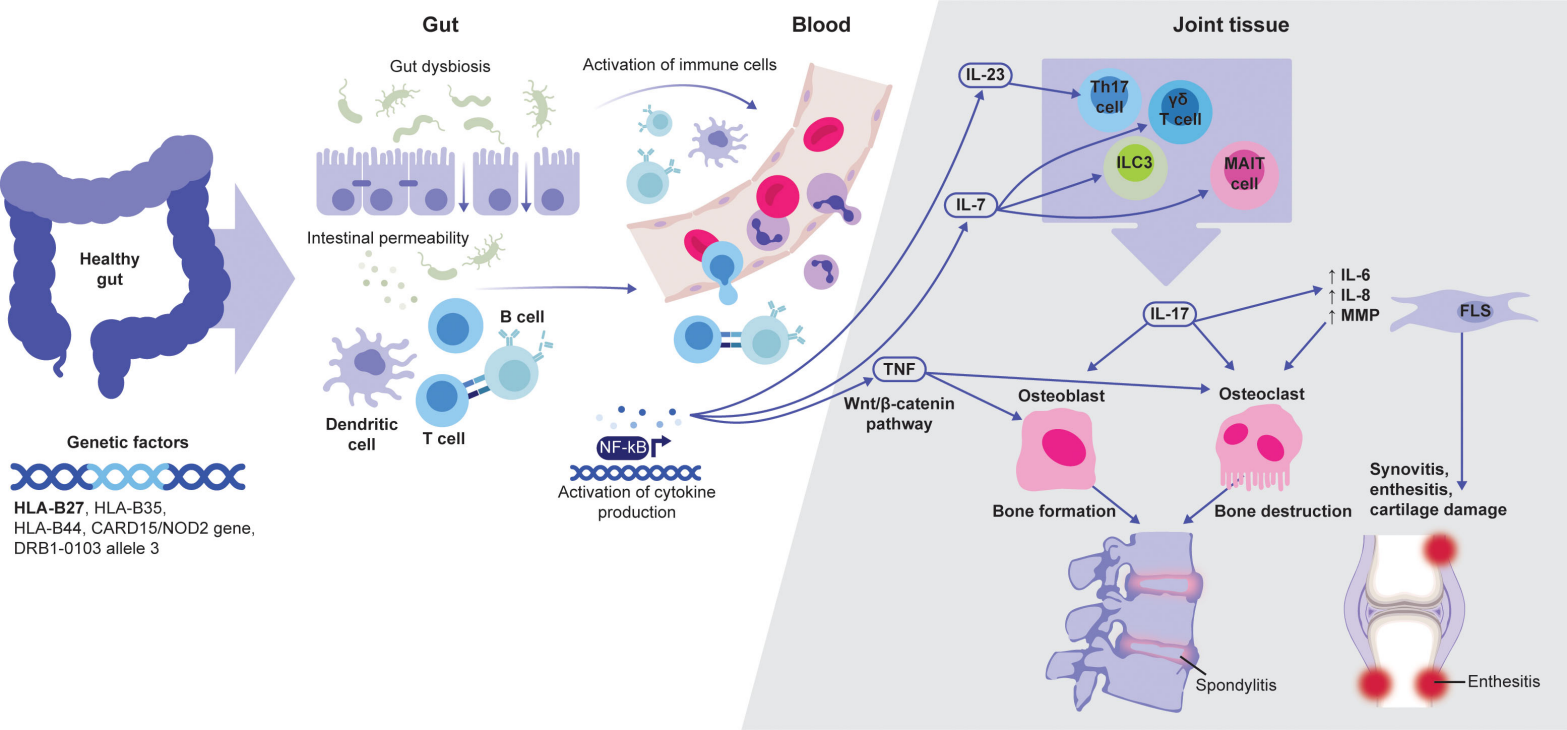
Schematic overview of the link between JAK-dependent cytokines and axSpA pathophysiology. Adapted from Felice C, et al. *Int J Mol Sci*. 2023;24:3957 (Copyright: ^©^ 2023 by the authors. Licensee MDPI, Basel, Switzerland; open access article distributed under the terms and conditions of the Creative Commons Attribution (CC BY) license [https://creativecommons.org/licenses/by/4.0/]). AxSpA development is associated with several genetic factors, of which HLA-B27 is the most prominent. Abnormalities and dysbiosis in the gut can lead to the activation of local immune cells. Subsequent cytokine production, including IL-7 and IL-23, further activate tissue-resident cells, such as MAIT, γδ T and Th17 cells, and ILC3. These cells can produce IL-17 that, together with TNF, play a major role in driving entheseal and bone inflammation, leading to axSpA-specific tissue damage. AxSpA, axial spondyloarthritis; FLS, fibroblast-like synoviocytes; ILC3, type 3 innate lymphoid cells; MAIT, mucosa-associated invariant T; NF-κB, nuclear factor-κB.

AxSpA inflammation is mediated and sustained by immune pathways, including cyclooxygenase-2, IL-17, and TNF as central drivers in disease development (**Figure 1**) (3). Recent studies also indicate a role for novel cells of the innate immune system in axSpA pathogenesis (19).

### 2.1 Pointers from genetics

AxSpA is highly heritable and polygenic, partially overlapping with other spondyloarthritis diseases (1). AxSpA is more common in first-degree relatives and other family members of patients with axSpA, and is highly associated with HLA-B27 (20). The presence of HLA-B27 is associated with early onset of axSpA symptoms, and predominant hip and sacroiliac joint involvement with reduced peripheral arthritis and dactylitis (21). HLA-B27 has a role in the presentation of bacterial antigens and self-antigens and activation and proliferation of expanded CD8^+^ T cell clonotypes found in circulation and affected tissues (21). Recently, Yang X et al. isolated, in patients with r-axSpA, clonotypes of CD8^+^ T cells bearing T-cell receptors (TCRs) with disease-specific β-chain sequences (22). Clonally-expanded TCR-BV9 β-chains were paired in r-axSpA with the AV21 α-chain (22). These TCRs responded to specific HLA-B*27:05-associated peptides corresponding to various microbial and self-antigens (22). These cross-reactive TCRs highlight a potential mechanism for HLA-B27 involvement in disease pathophysiology (22). Furthermore, misfolding of the HLA-B27 protein in the endoplasmic reticulum and the subsequent triggering of the unfolded protein response may also play a role in the pathogenesis of axSpA (23). Besides HLA-B27, other non-HLA susceptibility loci for axSpA have also been identified. Examples include single nucleotide polymorphisms (SNPs) in genes encoding the endoplasmic reticulum aminopeptidases (ERAP1 and ERAP2), enzymes involved in processing peptides to be presented by major histocompatibility complex (MHC) class I molecules (24) and other SNPs related to T-cell differentiation/function, including T-box transcription factor (Tbet) and Runt-related transcription factor 3 (Runx3) (25).

Several studies also identified associations between JAK-STAT pathway-related SNPs and rheumatic diseases; genetic variants of STAT4 and STAT3 were reported to be associated with susceptibility to axSpA (26–28), while SNPs of STAT4 and tyrosine kinase (TYK)2 may be associated with susceptibility to PsA (along with rheumatoid arthritis) (29).

AxSpA is genetically associated with several SNPs of the IL-23 receptor (IL23R) gene locus that may influence IL-23-driven IL-17 production, methylation of an enhancer region capable of promoting the differentiation of Th17 cells, possibly also through effects on IL23R or the IL-12R β2 subunit gene, which are adjacent (30). Recent genome-wide association studies identified several risk-associated SNPs in the prostaglandin EP4 receptor gene, providing strong evidence for a pathogenic role of prostaglandin E2 and its EP4 receptor in axSpA (31). A recent study demonstrated that EP4 is significantly overexpressed in Th17 cells of patients with axSpA where it may regulate IL-23R expression by suppressing forkhead box protein O1 (FOXO1), an inhibitor of retinoic acid receptor-related orphan receptor-γt (RORγt), thus enhancing STAT3 phosphorylation (32).

### 2.2 IL-17 and TNF inhibitor pathways

Among the proinflammatory pathways, TNF and IL-17 are of particular interest, as treatments that target these cytokines have been shown to be useful in axSpA therapy.

TNF is a potent proinflammatory cytokine and key orchestrator of systemic inflammatory and immune responses (33). TNF is produced by activated macrophages and monocytes in response to tissue damage or extracellular pathogens and other inflammatory triggers (34). It is also produced by innate and adaptive T cells. Pre-clinical/clinical evidence demonstrated that TNF is the main mediator of inflammation, tissue destruction, and cachexia associated with axSpA (35, 36). In all phases of structural damage in axSpA (inflammation, bone erosion, and bone formation), TNF plays a fundamental pathogenetic role (37). Elevated TNF concentration has been shown in inflamed sacroiliac joints of patients with AS, which is relevant to early disease stages, and TNF inhibitors (TNFi) effectively target inflammation (38, 39). The erosive stage of the disease can largely be attributed to the capacity of TNF to stimulate osteoclastogenesis (40, 41). The hallmark feature of r-axSpA – formation of new bone leading to ankylosis – is also influenced by TNF through activation of the wingless/β-catenin pathway, a regulatory pathway controlling osteoblast differentiation (42–44).

The IL-17 superfamily consists of six ligands (IL-17A to IL-17F), capable of binding to five subtypes of receptors (IL-17RA to IL-17RE) (45, 46). IL-17A is the prototype ligand and can signal as a homodimer or heterodimer with IL-17F (46). IL-17A and IL-17F signal through a dimeric IL-17RA and IL-17RC receptor, thus inducing the production of inflammatory cytokines and chemokines (45). IL-17A induces synovitis by stimulating fibroblast-like synoviocytes (FLS) to produce IL-6, IL-8, and matrix metalloproteinases (47). IL-17A also induces FLS proliferation and pannus formation and, together with IL-8, induces neutrophil activation (48–55). Furthermore, it can be produced by type 3 innate lymphoid cells (ILC3) and γδ T cells within entheseal tissue (56, 57). Produced locally, IL-17A can potentially amplify enthesitis by inducing cytokine production by resident mesenchymal cells (58) and affecting osteogenesis (59). IL-17 production has been linked with IL-23: in animals, IL-23 was shown to be a potent activator of Th17 cells, and IL-23 inhibition often led to similar effects as IL-17 inhibition (60), including efficacy in psoriasis (61).

## 3 JAK signaling and inhibition

JAK inhibition represents a novel mechanism by which chronic inflammation can be controlled, acting intracellularly, downstream to the cytokine inhibitors (14). JAKi are effective in a range of systemic inflammatory diseases, including axSpA (62). JAKs are a family of intracellular TYKs that facilitate the signaling process of >50 cytokine receptors (63). Individual JAK enzymes associate with the intracellular domains of receptor subunits of the class I and II receptor superfamily, comprising two large classes of single pass transmembrane-domain-containing receptors employed by a broad range of cytokines and growth factors (14). Cytokines initiate signaling by binding to extracellular domains of receptors, inducing multimerization of receptor subunits (14). This brings the non-covalently associated JAKs proximal to one another, resulting in phosphorylation and activation of STAT proteins. A phosphorylated STAT dimer then translocates to the nucleus to initiate transcription of cytokine-responsive genes (64).

**Figure 2:**
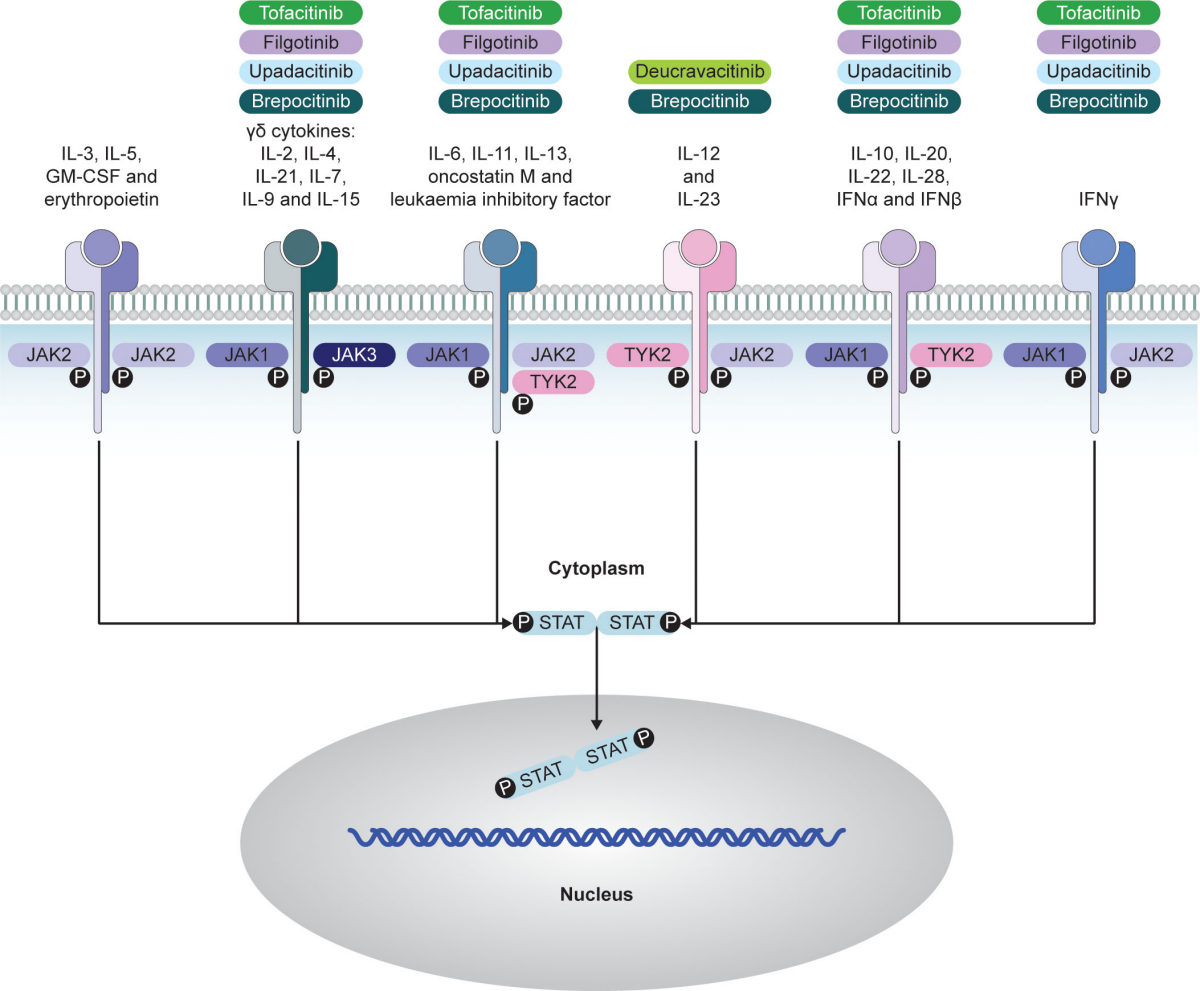
JAK–STAT-dependent transmission of cytokine signaling and respective JAKi. Adapted from Choy EH. *Rheumatology.* 2018;58(6):953-962 (^©^ The Author[s] 2018. Published by Oxford University Press on behalf of the British Society for Rheumatology; open access article distributed under the terms of the Creative Commons Attribution License [http://creativecommons.org/licenses/by/4.0/], which permits unrestricted reuse, distribution, and reproduction in any medium, provided the original work is properly cited). GM-CSF, granulocyte–macrophage colony-stimulating factor; IFN, interferon; IL, interleukin; JAK, Janus kinase; JAKi, Janus kinase inhibitors; P, phosphorylation, STAT, signal transducer and activator of transcription; TYK, tyrosine kinase.

There are four members of the JAK family: JAK1, JAK2, JAK3, and TYK2, all functioning in pairs (14). There is a high degree of sequence homology across the JAK family, with the highest homology observed within the adenosine triphosphate (ATP)-binding site (65). Different JAKs are linked to cytokine receptors, and specific signals depend on the dominance of one JAK over another in pairings (66). JAK1 pairs with three other JAKs, regulating various cytokine receptors, including IL-6 and type I interferons (IFNs) (66). JAK2, unique in self-pairing, is crucial in growth factor signaling (66). JAK1 or JAK2 deficiency is generally incompatible with life (64). JAK3 and TYK2 mediate a smaller number of signaling pathways, and human deficiencies are less severe, with effects predominantly being on the arms of the immune system and/or associations with specific bacterial and viral infections (66) (**Figure 2**).

JAKi such as tofacitinib, upadacitinib, and filgotinib target the kinase domain and inhibit by competing with ATP at the catalytic site in its active confirmation, which is induced upon cytokine binding to its receptor (67, 68). Others are allosteric inhibitors, such as deucravacitinib, which inhibits TYK2 via the JH2/pseudokinase domain (69). JAKi have become important medicines in various inflammatory diseases and myeloproliferative disorders (67).

While most JAKs are expressed ubiquitously, JAK3 is expressed in hemopoietic lineages and vascular muscle cells (66). As the JAK–STAT pathway mediates signaling of multiple proinflammatory cytokines (66), this introduces a high level of complexity. Although particular JAKi have demonstrated efficacy in axSpA, unraveling the exact mechanisms of their therapeutic effect represents a considerable challenge. Another particular challenge is the complexity of JAK-STAT signaling and the potential importance of selective inhibition of particular JAKs over others; this is further discussed below.

## 4 Mechanistic rationale for JAK inhibition in axSpA

Beyond the established role of TNF and IL-17, recent evidence supports the involvement of JAK–STAT-related cytokines. While bDMARDs target single cytokines, JAK inhibition blocks multiple pathways (66). Two JAKi (tofacitinib and upadacitinib) were investigated in patients with axSpA and inadequate response to NSAIDs; however, these trials also included some patients with inadequate response to TNFi (10, 70). In all clinical trials, the primary endpoints were met and JAKi were efficacious versus placebo (10, 70, 71).

JAKi were efficacious against axial symptoms, significantly reduced pain, and improved function, fatigue, quality of life, and other patient-reported outcomes (10, 70, 72). To date, there are no head-to-head trials comparing the efficacy of JAKi with TNFi or IL-17 inhibitors, although efficacy appears comparable both within and between classes of advanced therapy in axSpA (3). bDMARDs are the first line of advanced therapy used in the treatment of axSpA; however, loss of response can occur due to intolerance or treatment failure, with the formation of neutralizing anti-drug antibodies (ADAs) representing a cause of secondary treatment failure (73). JAKi have been shown to be effective and safe among patients with prior exposure to bDMARDs. Upadacitinib efficacy was demonstrated through 104 weeks among patients with AS and inadequate response/intolerance to bDMARDs (74). Likewise, tofacitinib was demonstrated to be efficacious in bDMARD-naïve and TNFi-inadequate responder patients with AS (75). As JAKi are small molecules, they are not expected to induce ADA formation; however, adverse events or loss of response may result in discontinuation. While there are limited clinical data demonstrating the efficacy of JAKi switching in axSpA, small observational studies of patients with rheumatoid arthritis found that treatment with a second JAKi was safe and effective after discontinuation of the first JAKi (76, 77).

In studies testing JAKi in axSpA, safety appeared comparable with other diseases (10, 70, 71). Although JAKi efficacy in axSpA was demonstrated in multiple clinical studies (10, 70, 71); it remains unknown which patients have the highest chance of benefiting from JAKi therapy. Similar to other DMARDs and other rheumatic diseases, treatment with advanced therapies in patients with early active axSpA may represent an ideal opportunity to achieve treatment response and increase probability of remission (78). Indeed, the ESTHER and INFAST studies found that rates of remission reached over 40% among patients with early active axSpA of <5- or 3-years’ duration, respectively (79, 80). This is more than double the remission rate among patients with advanced disease (81–83). Further insight regarding efficacy of JAKi in early axSpA is likely to be provided by future studies, including ToFAcitinib in Early Active Axial SpondyloarThritis: (FASTLANE), a phase 4 randomized, double-blind, placebo-controlled study designed to compare the efficacy and safety of tofacitinib in patients with early active axSpA (≤2 years) (NCT06112665; 84).

Overall, JAKi represent a potent drug class in axSpA with the ongoing need for better patient profiling likely based on the immunology behind the disease.

## 5 Rationale for the impact of JAKi on the gut–joint axis in axSpA: role of ILC3, γδ T cells, mucosa-associated invariant T cells, and tissue-resident memory T cells

As JAKi do not directly inhibit TNF or IL-17 signaling, the mechanisms underlying their therapeutic effects are not fully understood. Historically, it was hypothesized that the efficacy of JAKi in axSpA was due to JAK2 blockade, blunting IL-23 signaling (85, 86) and thus the IL-23/IL-17 axis and Th17 cells, which play a role in axSpA. However, this mechanism does not fully explain their therapeutic effect. Upadacitinib and tofacitinib demonstrate limited affinity to JAK2 versus other JAKs (87), and sole IL-23 inhibition lacks efficacy in axSpA (16). This shifts the focus to other potential IL-17-dependent mechanisms. Importantly, IL-17 can be produced independently from IL-23, in particular, by γδ T cells primed by IL-7, a JAK1-dependent cytokine (88). Other IL-7-dependent cell types include mucosal-associated invariant T (MAIT) cells and type 3 innate lymphoid cells (ILC3), which also produce IL-17 (89, 90).

The discovery of the importance of type 3 immunity (mediated by cells producing IL-17A and IL-17F) in axSpA pathogenesis led researchers to further explore relevant cytokines and cell subsets. Evidence supports a key role for innate immunity in driving the inflammatory processes of axSpA (25). ILC3, MAIT cells, and γδ T cells have been shown to be the major sources of IL-17A, IL-17F, and other inflammatory cytokines in axSpA (25). Of interest, these cells are of mucosal, and potentially intestinal, origin; however, ILC3 and γδ T cells are also present in the entheses of healthy individuals (25, 57, 91). An external factor, such as biomechanical stress, could trigger these cell subsets to induce inflammation in entheses (57, 91). This inflammatory process can self-resolve in healthy individuals, but in the presence of a genetic susceptibility (e.g. HLA-B27) and bacterial products translocated from the intestine, this process could self-perpetuate by inducing the typical axSpA inflammation (92). Together, this suggests a link between the gut and joints, or the so-called gut–joint axis, in axSpA. Intriguingly, many of these cells do not depend exclusively on IL-23 to produce effector inflammatory cytokines (25).

MAIT cells are innate immune cells that produce IFNγ, TNF, IL-2, IL-17A, and IL-22, and are characterized by their dependence on the MHC class I-related monomorphic protein MR1 for their selection and activation (93). MAIT cells have been demonstrated to be dysregulated in patients with axSpA, and can accumulate in inflamed joints, with their activation dependent on IL-7 but not IL-23 (89). IL-7 and IL-15 may be the most important cytokines, together with IL-23, in the expansion and activation of axSpA-associated innate immune cell subsets (94). Importantly, IL-7 and IL-15 depend on JAK1 and JAK3 for their signaling (67). Thus, JAK1 and JAK3 inhibitors could potentially exert their beneficial effects on axSpA disease activity through inhibiting IL-7- and IL-15-mediated activation of MAIT cells, independently of IL-23 (95).

In axSpA, dysregulated T-cell subsets might have a pathogenic role. Of particular interest are the tissue-resident memory T (T_RM_) cells, responsible for frontline protection against pathogens and tumor outgrowth at the level of mucosal and other epithelia (96). While CD8^+^ T_RM_ cells expand in the intestine, these cells can migrate outside the mucosal sites (97) and have been shown in the peripheral blood and synovial fluid of patients with axSpA (98–100). Although it has been suggested that CD8^+^ T_RM_ cells could migrate between gut and joints in axSpA (99), conclusive evidence is lacking, and further studies are required. CD8^+^CD103^+^ T_RM_ cells are activated, and produce cytokines, perforin, and granzyme B and their fate may depend on JAK-STAT signaling, as demonstrated by the ability of tofacitinib to suppress their functions in murine lupus nephritis (62). IFNα, IFNβ, IL-7, and IL-15 are involved in CD8^+^ T_RM_ cell formation (101).

This evidence suggests a rationale for a gut–joint–spine axis in axSpA, implying that alterations of the intestinal microbiome and permeability lead to aberrant activation of innate immune cells at the intestinal mucosa. These activated cells enter the systemic circulation and circulate to typical sites of axSpA inflammation (102). Aligned with this hypothesis, dysbiosis, altered intestinal permeability, systemic circulation of bacterial products, and the gut–joint recirculation of innate immune cells have been demonstrated in patients with axSpA (25). Cell-activating cytokines such as IL-7 and IL-15 depend on JAK1, supporting the potential of JAKi in axSpA, which is further supported by their efficacy in IBD. Nevertheless, direct support for this hypothesis of the mechanisms of action of JAKi in axSpA awaits further experiments (103–105).

## 6 Insights on JAK1 inhibition efficacy from pre-clinical models of axSpA

Despite decades of research, few axSpA animal models exist, and they only partially recapitulate human axSpA in its complexity. While only mimicking certain aspects of the disease, insights from these models could shed light on JAKi efficacy in axSpA.

**Figure 3:**
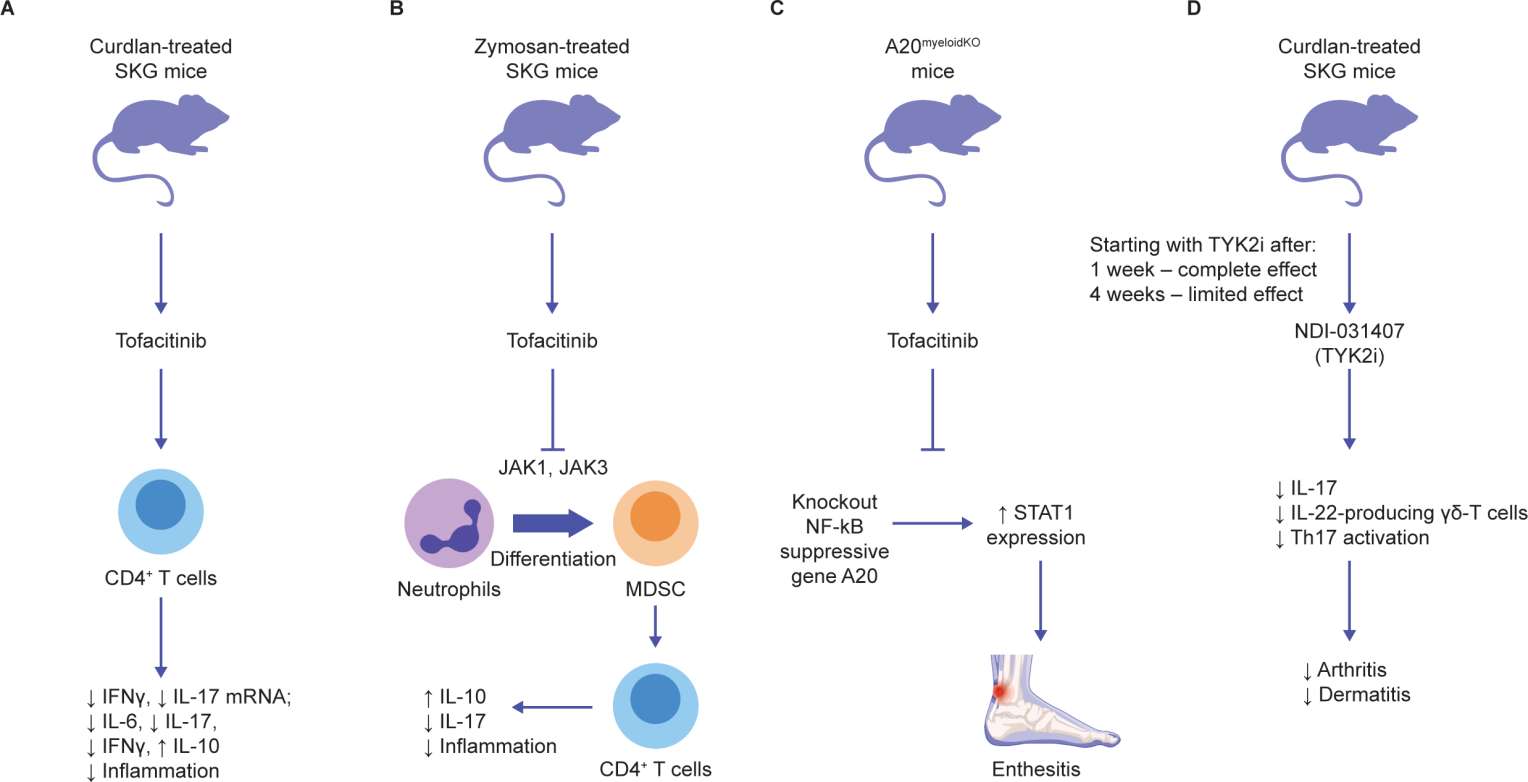
Effects of JAK inhibition on pathophysiology of axSpA animal models **(A-D)**. Schematic overview of the prominent animal studies with JAKi in animal models of axSpA and their main findings (further description can be found in the text) (2–5). AxSpA, axial spondyloarthritis; JAK, Janus kinase; JAKi, Janus kinase inhibitors; MDSC, myeloid-derived suppressor cell; mNRA, messenger ribonucleic acid; NF-κB, nuclear factor-κB; STAT1, signal transducer and activator of transcription 1; TYK2i, tyrosine kinase 2 inhibitors.

In curdlan-treated SKG mice, a model of spondyloarthritis, tofacitinib suppressed disease progression to a similar extent when administered after disease onset (clinical score 4/6) for either 14 or 28 days (average score 2) (**Figure 3A**) (106). Reduced disease severity was associated with reduced IL-17 and IFNγ production by CD4^+^ T cells in the lymph nodes and spleen. In joint tissue, reduced *Il6*, *Il17*, and *Ifnγ* mRNA and increased *Il10* mRNA were observed. The prolonged effect of tofacitinib, even 2 weeks after dosing was finished, and IL-10 induction suggest a potential tolerogenic effect (106).

In another study, zymosan-treated SKG mice were treated with tofacitinib for 2 weeks, resulting in reduced joint inflammation and enthesitis (**Figure 3B**) (107). Myeloid-derived suppressor cells (MDSCs), a heterogeneous group of cells that suppress T-cell responses, accumulated in the bone marrow and spleen of SKG mice after zymosan (107). This was further increased after tofacitinib was dosed from day 0 to 42, while arthritis was completely abrogated (107). Adoptive transfer of MDSCs from 1 week after zymosan ameliorated SKG arthritis (107). The anti-arthritic effect of tofacitinib was reversed with neutrophil depletion. Addition of tofacitinib to granulocyte-macrophage colony-stimulating factor (GM CSF)-treated bone marrow myeloid cells from SKG mice was found to facilitate MDSC differentiation and reduce myeloid dendritic cell (DC) differentiation *in vitro* (107). These data suggest that tofacitinib reduces immunogenic DCs and enhances the differentiation of neutrophil-derived MDSCs; these MDSCs influence CD4^+^ T cells to enhance IL-10 production, thus downregulating inflammation.

The role of the JAK–STAT1 pathway in spondyloarthritis was further demonstrated in mice wherein the nuclear factor-κB (NF-κB) regulatory *A20* gene was knocked out in myeloid cells (**Figure 3C**) (6). These mice developed Achilles enthesitis, which was partially suppressed by tofacitinib (6). *In vitro*, A20 expression partially suppressed IL-6-induced STAT1 but not STAT3 expression (6). These data suggest that tofacitinib suppresses enthesitis driven by innate immune inflammatory pathways, but not the low-level disease driven locally by the response to mechanical stress (108). Furthermore, some of the function of A20 involves regulation of STAT1, in addition to its major regulatory action on NF-κB (6). Thus, the profound impact of tofacitinib on entheseal inflammation may relate to secondary impacts on cytokine-driven suppression of NF-κB.

Together, these data suggest that JAK1 inhibition is likely to act via a broad range of cells/pathways, abrogating multiple inflammatory loops and resulting in the suppression of the IL-17 and TNF key pathways.

## 7 JAK selectivity and new approaches

**Figure 4:**
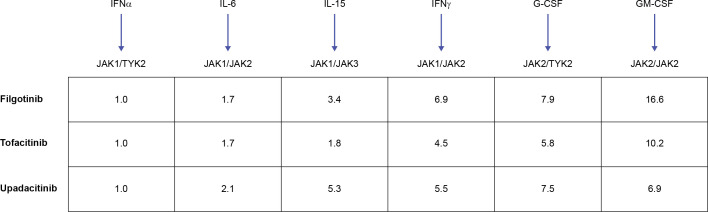
JAKi cellular selectivity for JAK heterodimeric cytokine signaling. Mean fold JAKi selectivity of each JAK pair versus inhibition of JAK1/TYK2 pathway in monocytes. A higher value denotes higher selectivity versus JAK1/TYK2-dependent signaling. Adapted from Traves PG. *Ann Rheum Dis.* 2021;80:865-875 (^©^ The Author[s] [or their employer(s)] 2021. Published by BMJ; open access article distributed in accordance with the Creative Commons Attribution Non Commercial License (CC BY- NC 4.0; http://creativecommons.org/licenses/by/4.0/), which permits others to distribute, remix, adapt, build upon this work non-commercially, and license their derivative works on different terms, provided the original work is properly cited, appropriate credit is given, any changes made indicated, and the use is non-commercial). G-CSF, granulocyte colony-stimulating factor; GM-CSF, granulocyte–macrophage colony-stimulating factor; IFN, interferon; IL, interleukin; JAK, Janus kinase; JAKi, JAK inhibitor; TYK, tyrosine kinase.

JAK selectivity has generated much attention since the implementation of the JAKi in research and care. Due to the paired functioning of JAK enzymes and overall complexity of JAKi biology, understanding of selectivity can be obtained through rigorous mechanistic, pharmacologic, and metabolic research, and clinical evaluation. The available JAKi (including tofacitinib, upadacitinib, and filgotinib, which are used/investigated in axSpA) potently inhibit JAK1 with varying effects on JAK3 and JAK2 (87, 109). Theoretically, more selective JAKi could provide more targeted treatment and avoid adverse effects. These theoretical benefits are most likely to emerge from avoiding JAK2-dependent effects, which include the production of red blood cells and platelets (109, 110), although the JAK2-mediated effect on IL-23 may also benefit patients with axSpA. However, JAK1 selectivity remains inherently broad: JAK1 pairs with JAK3 and TYK2, thus JAK1 inhibition targets a broad range of cytokines (109, 111). This selectivity also may be to an extent dose-dependent *in vitro* (87). The translation of these differences to the clinical setting and the link between JAK selectivity and the efficacy and safety profile of individual JAKi remains a considerable challenge (111). Nevertheless, all JAKi showing efficacy in axSpA have a strong effect on JAK1 and it appears intriguing whether selectivity beyond JAK1 is likely to yield differing clinical results. JAKi cellular selectivity (as based on inhibition of cytokine signaling via different JAK pairings) is summarized in **Figure 4**, showing that JAKi used or investigated in axSpA have >5-fold selectivity for JAK1 versus JAK2-dependent signaling.

TYK2- and JAK3-selective JAKi appear to have a limited effect on JAK1 and appear to be distinct from the existing JAKi options. TYK2, also a JAK, mediates signaling downstream of type I IFN, and the IL-10/-22 and IL-12/-23 receptor families (112). *TYK2* loss-of-function genes are associated with protection against axSpA (113), and TYK2 deficiency may lead to increased susceptibility to mycobacterial/viral infections (114). In T cells, the TYK2 inhibitor, NDI-031407, blocked IL-23R but not IL-6R-mediated STAT3 (115). Conversely, tofacitinib and ruxolitinib blocked both IL-23R and IL-6R (115). Interestingly, the TYK2 inhibitor completely blocked peripheral and axial disease in SKG mice if administered 1 week after administration of the disease trigger, curdlan (**Figure 3D**) (115). However, 4 weeks after the disease was triggered, broadly acting tofacitinib, but not the selective TYK2 inhibitor, could suppress peripheral disease (115). Both drugs comparably suppressed spondyloarthritis when initiated 4 weeks after the disease was triggered (115). The allosteric inhibitor deucravacitinib also suppressed IL-23-mediated IL-17 production by CD4+ T cells in pre-clinical models of colitis, psoriasis, and IFN-mediated lupus (69, 116). These data support the hypothesis that inhibitors that block IL-23 and its downstream effects are most effective in preclinical models of spondyloarthritis early in the disease process, which is dominated by IL-23. However, JAKi with an effect on JAK1, including tofacitinib and upadacitinib, continue to be effective later in the disease, as secondary proinflammatory pathways assume greater importance. In phase 2 trials of deucravacitinib in PsA, 75% of patients achieved a ≥75% Psoriasis Area and Severity Index improvement from baseline (PASI75) and 63% achieved a ≥20% improvement in American College of Rheumatology (ACR) response criteria (13, 117).

To date, deucravacitinib has demonstrated efficacy in PsA (13) and psoriasis (118, 119). Currently available JAKi have a strong effect on JAK1 and limited effect on JAK2, which differs from TYK2 inhibition. Thus, deucravacitinib is likely to exert its effect mainly by inhibiting JAK2/TYK2-mediated IL-23 signaling and subsequently IL-17 production (120, 121), which is closely linked with psoriasis and PsA pathophysiology. Deucravacitinib clinical findings are complemented by an acceptable safety profile, however, current data lack long-term follow-up (13, 118, 119).

Another JAKi in clinical development with limited JAK1 effects is ritlecitinib, which uniquely and selectively binds JAK3 via a covalent (i.e., irreversible) interaction in the JH1/TYK domain (122, 123). Ritlecitinib also inhibits the Tec family of kinases, which includes Bruton’s tyrosine kinase and IL-2-inducible T cell kinase (122, 123). Long-term studies are needed to understand the risk/benefit profile of selective JAK3/Tec inhibition.

Brepocitinib (PF-06700841) is a TYK2/JAK1 inhibitor, which suppressed IL-23 *in vitro* and adjuvant arthritis *in vivo* (124). A phase 2b trial showed that brepocitinib was superior to placebo in reducing signs and symptoms of PsA (125). In a phase 2 trial of patients with psoriasis, 80% of patients achieved PASI75, and C-reactive protein level was reduced by 50% (126). It remains to be investigated to what extent efficacy and safety profiles of brepocitinib differ from the JAKi options already used in clinical practice.

To the best of our knowledge, the effects of deucravacitinib, ritlecitinib, and brepocitinib have not been studied in axSpA pre-clinical models or patients with axSpA.

## 8 Conclusion

Emerging research helps deepen the understanding of axSpA pathophysiology. In addition to the fundamental research, clinical trials provide important insights into disease pathophysiology, demonstrating the efficacy of some pharmaceutical agents and failure of others. Exemplifying this process, several JAKi show efficacy in a substantial proportion of patients with axSpA. JAKi block a range of cytokines and pathways, while not focusing directly on TNF and IL-17, traditionally seen as the main disease drivers in axSpA. Nevertheless, these results, together with the recent developments in the fundamental research, highlight the potential importance of understudied cell types, such as ILC3, γδ T cells, neutrophil-derived MDSCs, and cytokines, including IL-7. These pathways may link mucosal inflammation with IL-17 production that further escalates disease at the entheses and bone.

Further research into these cell types and pathways, along with more selective JAKi, including those with less JAK1-mediated effects, could expand the arsenal of effective medicines, and improve our understanding of how bacteria interact with the immune system of genetically at-risk individuals to trigger disease development.
